# Flame-Retardant Ionic Conductive Elastomers with Multiple Hydrogen Bonds: Synthesis, Characterization, and Strain Sensing Applications

**DOI:** 10.3390/molecules30081810

**Published:** 2025-04-17

**Authors:** Sen Li, Hao Chen, Chen Zhao, Jinlin He, Lijing Zhang

**Affiliations:** 1 College of Safety Science and Engineering, Nanjing Tech University, Nanjing 210009, China; lisen321283@163.com (S.L.); zhanc573@gmail.com (C.Z.); 2 College of Chemistry, Chemical Engineering and Materials Science, Soochow University, Suzhou 215123, China; chenhao00110@163.com

**Keywords:** flame-retardant polymer, ionic conductive elastomer, hydrogen bonds, strain sensor

## Abstract

Flammability is a significant challenge in polymer-based strain sensing applications. In addition, the existing intrinsic flame retardant is not elastic at room temperature, which may potentially damage the flexible equipment. This study presents a series of flame-retardant ionic conductive elastomers (ICEs) (denoted as PCAIP_x_) containing phosphorus from phytic acid (PA) and nitrogen from choline chloride (ChCl) with multiple hydrogen bonds synthesized using a simple and efficient one-pot UV-initiated radical copolymerization of a polymerizable deep eutectic solvent (PDES). The limiting oxygen index (LOI) value increased from 24.1% for the pure PCAI without PA to 38.3% for PCAIP_7.5_. The SEM analysis of the residual char shows that the formation of the dense and continuous char layer effectively worked as a shield, preventing further decomposition of the undecomposed polymer inside while hindering the transmission of heat and mass and isolating the oxygen required for combustion. The hydrogen bonds’ cross-linked structure and phosphorus-containing elastomer demonstrate a superior elasticity (elongation at break of up to 2109%), durability, and tear resistance and excellent adhesive properties. Application of PCAIP_X_ in strain sensors showed that the elastomer has excellent cyclic stability and exhibited repeatable and stable resistance change signals in response to repetitive bending motions of the wrist, fingers, elbow, and knee. Consequently, this study provides a simple strategy for the development of a flame-retardant ICE which can effectively reduce fire hazards and potentially be applied in other fire-risk fields such as personal protection, firefighting, and sports equipment.

## 1. Introduction

Polymeric materials, renowned for their superior performance, are extensively utilized across industrial and domestic applications. However, their innate flammability also renders them a common fuel source in contemporary fires, particularly within the contexts of architectural and urban conflagrations [1,2,3,4]. The combustion behavior of these materials is characterized by unique complexities, encompassing thermal behavior alterations, pyrolytic chemical reaction sequences, and the combustion process itself [5,6,7,8,9]. Under fire conditions, polymers frequently exhibit distinctive changes such as melting, foaming, expansion, and contraction, which significantly influence the pyrolysis, ignition, and combustion processes [10,11,12,13,14,15]. Moreover, the pyrolytic reaction mechanisms of polymers may be altered by various factors present in fires, including high heat fluxes [16,17,18,19,20]. Despite the extensive research conducted by scholars worldwide on the combustion behavior of polymers in fires, the interplay of chemical reactions, mass transfer, and heat transfer poses significant challenges and opportunities in efforts to mitigate the fire risks associated with polymers through flame-retardant treatments [21,22,23,24,25,26,27,28,29].

The mechanisms of flame retardancy are typically categorized into gas-phase flame retardation, condensed-phase flame retardation, and heat exchange interruption mechanisms [12,19]. The heat exchange interruption mechanism primarily operates through physical means, utilizing the endothermic degradation of chemical substances to reduce temperature. The gas-phase flame retardation mechanism is chemical in nature, functioning by generating an increased volume of non-combustible gases to dilute the oxygen concentration, thereby preventing the combustion of materials. Similarly, the condensed-phase flame retardation mechanism is chemical based, involving the production of non-combustible gases and char by chemical substances, which form a physical barrier to impede heat and mass transfer between the gas and condensed phases. Flame retardants, which are substances used to impede the ignition or spread of combustion in materials, can be broadly classified into several categories based on their chemical composition and mechanisms of action. These include halogenated flame retardants, silicone-based flame retardants [5], phosphorus-based flame retardants [10], nitrogen-based flame retardants [20], and inorganic flame retardants [13]. Notably, halogenated flame retardants have been progressively phased out due to the release of toxic gases (HCl or HBr) upon combustion. There is a growing demand for flame retardancy that surpasses the capabilities of single-element agents, leading to a significant interest in the development of multi-element synergistic flame-retardant systems. These systems, which integrate two or more components, aim to achieve a more effective fire-resistant outcome than the sum of their individual effects, offering superior comprehensive performance [5,9,10].

In recent years, bio-based flame retardancy has attracted extensive attention from researchers, and a series of bio-based flame-retardant materials has been prepared [30,31,32]. Phytic acid (PA) is a bio-based molecule which is mainly found in the seeds, roots, and stems of plants, and is a natural resource that can be recycled. PA has a high content of the flame-retardant element phosphorus (with 6 negatively charged phosphate groups connected to 12 hydroxyl groups that can be hydrolyzed into hydrogen ions) and a highly symmetrical molecular structure. Under acidic conditions, it can chelate with metal cations that can catalyze the formation of carbon, and generate stable and non-hydrolytic complexes, which have very excellent flame-retardant potential [18,33]. Because phytic acid has excellent physiological and chemical properties, it has been widely used as an important organophosphorus additive in food, medicine, and other fields; however, the time spent on formal research on its application in the field of flame retardants is relatively short, and its huge potential is still waiting for people to tap. You et al. presented an intrinsic flame-retardant bio-based elastic phytic acid polyurethane (PUPA) with PA and diglycerol synthesized using simple and efficient one-pot polycondensation [33]. The cross-linked structure and polar phosphorus-containing segments of PUPA are fabricated into PUPA-TENG, demonstrating a superior elasticity, flame retardancy, impact resistance, and dielectric constant. Consequently, this study provides a simple strategy for tailoring TENGs toward environmentally friendly and secure power generators and electronics, which can effectively reduce fire hazards and potentially be applied in other fire-risk fields such as personal protection, firefighting, and new energy. Zhang et al. prepared a new type of flame-retardant polyelectrolyte complex (PEC) by using PA and polyethylenimide (PEI) [18]. It was found that, when 20 wt% PA, PEI, and PEC were added to PP matrix, their oxygen indices reached 19.8 vol%, 18.9 vol%, and 25.1 vol%, respectively. The introduction of PEC significantly improved the carbonization degree of PP during combustion and effectively controlled the generation of combustible gas during combustion, achieving an excellent flame-retardant effect.

An elastomer is a kind of polymer with excellent mechanical properties, certain strength, and other characteristics which is widely used in electrical conductors, human body functional equipment, and other applications. However, with the frequent occurrence of fire and other accidents, flame-retardant elastomers are the inevitable direction of our future development [34,35,36,37,38,39,40,41,42,43]. Among them, ionic conductive elastomers (ICEs) are unique materials within elastomers that possess excellent electrical conductivity. Benefiting from their good flexible properties, the applications of ICEs in flexible sensing have witnessed rapid development [44,45,46,47]. In a recent piece of research, we prepared an ICE material using the copolymerization of a ternary polymerizable deep eutectic solvent (PDES) containing choline chloride (ChCl), hydroxyethyl acrylate, and itaconic acid (IA). The resulting ICE exhibited excellent mechanical properties and demonstrated good sensing characteristics when applied in strain sensors [48]. The objective of this study is to develop a flame-retardant ICE (PCAIP_X_) with multiple hydrogen bonds derived from ChCl and PA with adjustable chemical structures and phosphorus content by controlling the adding of the monomer and PA. The hydrogen bonds’ cross-linked structure and phosphorus-containing elastomer are flame retardant and demonstrate superior elasticity, durability, and tear resistance and excellent adhesive properties and performance. Therefore, PCAIP_X_ has significant potential as an easy-to-use, safe, and environmentally friendly strain sensing material which can be applied in firefighting and personnel protection equipment.

## 2. Results and Discussion

**Scheme 1:**
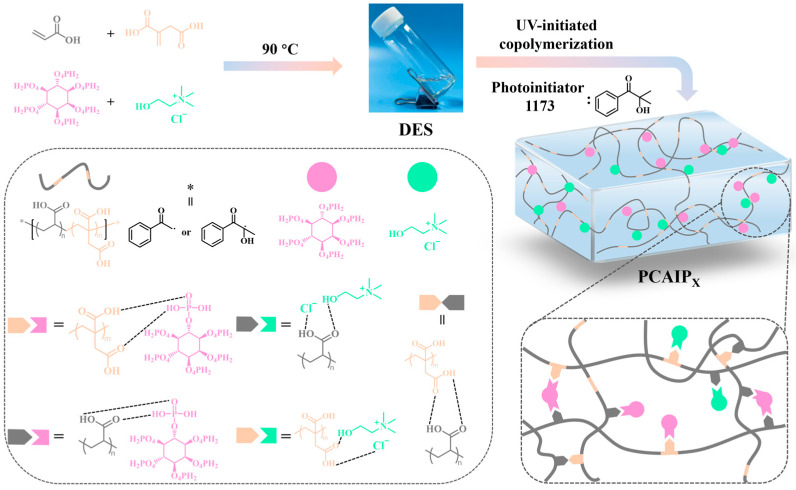
Schematic diagram of the synthesis of a flame-retardant ICE with multiple hydrogen bonds.

As shown in Scheme 1, the AA, IA, ChCl, and PA components were mixed at 90 °C to obtain a transparent PDES named CAIP_X_ (X = 5, 7.5, 10, 12.5, indicating the weight ratio of PA), which was then added to a minimum photoinitiator and exposed to UV irradiation to yield the ICE materials named PCAIP_X_. In the PCAIP_X_ materials, the various components are rich in hydrogen bonds, which endow them with favorable mechanical properties and adhesion. Additionally, the nitrogen-containing component ChCl and the phosphorus-containing component PA can impart certain flame-retardant behavior to the material.

### 2.1. Structure Characterization of CAIP_X_ and PCAIP_X_

**Figure 1:**
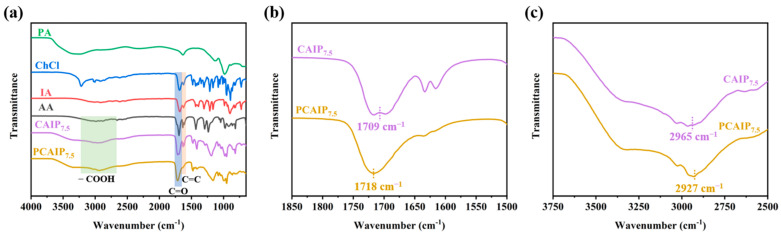
(**a**) FTIR spectra of PA, ChCl, IA, AA, CAPI_7.5_, and PCAPI_7.5_, (**b**,**c**) the enlarged FTIR figures of CAPI_7.5_ and PCAPI_7.5_.

Four phosphorus–nitrogen-containing CAIP_X_ PDESs with adjustable chemical structures and phosphorus content are synthesized. The chemical structures of typical CAIP_X_ and PCAIP_X_ were verified using FT-IR (Figure 1). It can be clearly seen that the carbon–carbon double bond disappeared at 1600 cm^−1^ after the polymerization, while the peak of carboxyl at 2965 cm^−1^ shifted to 2927 cm^−1^ and the peak of carbonyl shifted to 1718 cm^−1^ from 1709 cm^−1^ after polymerization, proving the successful synthesis of the polymer [25,49,50].

**Figure 2:**
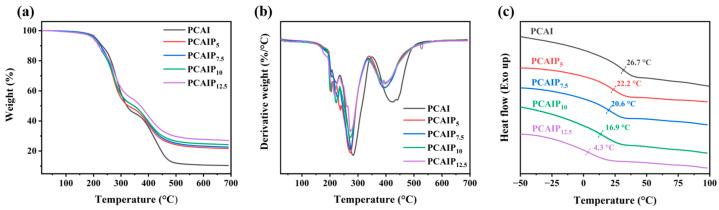
(**a**) TGA, (**b**) DTG, and (**c**) DSC curves of PCAI and PCAIP_x_.

The thermal properties of PCAI without PA and PCAIP_X_ with different PA contents were characterized via TGA and DSC techniques and the results are shown in Figure 2. The initial decomposition temperature (*T_i_*), maximum thermal decomposition temperature (*T_max_*), and residual weight of PCAI were 208.7 °C, 282 °C, and 10.4%. With the addition of PA, the *T_i_* of PCAIP_X_ exhibited a slightly decreasing trend. Specifically, the *T_i_* of PCAIP_5_ decreased to 205.8 °C, that of PCAIP_7.5_ decreased to 204.5 °C, that of PCAIP_10_ decreased to 203.6 °C, and that of PCAIP_12.5_ decreased to 203.1 °C. Similarly, the *T_max_* of PCAIP_5_ decreased to 272.3 °C, for PCAIP_7.5_, it was 272.4 °C, for PCAIP_10_, it reached 272.3 °C, and, for PCAIP_12.5_, it decreased to 272.5 °C. However, the residual weight increased from 10.4% of the PCAI to 21.6%, 22.6%, 24.26%, and 27.0% of PCAIP_5_, PCAIP_7.5_, PCAIP_10_, and PCAIP_12.5_, respectively, illustrating that the adding of PA increased the carbonization of PCAIP_X_. Additionally, the *T_i_* of the resultant PCAIP_X_ gradually decreased with the increase in PA. At the same time, the residual weight gradually increased, which was mainly related to the increase in P content. The presence of minor peaks preceding *T_max_* in the DTG curve proved the occurrence of ester exchange reactions (Figure 2b). For the residual mass at 282 °C, the adding of PA significantly increased the residual mass of PCAIP_X_ in comparison with PCAI, which signified that the PA in PCAIP_X_ possessed the capability to catalyze the char formation of PCAI during thermal degradation, thus holding promise for enhancing the charring ability of PCAIP_X_. Figure 2c shows the DSC curves of PCAI and PCAIP_X_. For PCAI, the *T_g_* reached 26.7 °C. Figure 2c reveals a discernible decline in the *T_g_* of PCAIP_X_ upon the incorporation of PA; the *T_g_* of PCAIP_5_, PCAIP_7.5_, PCAIP_10_, and PCAIP_12.5_ signally decreased to 22.2, 20.6, 16.9, and 4.3 °C, respectively. This may be attributed to the increased water content and weakened intermolecular interactions that accompany the rise in phytic acid levels, which, in turn, facilitate greater molecular mobility.

### 2.2. Flame-Retardant Property

**Figure 3:**
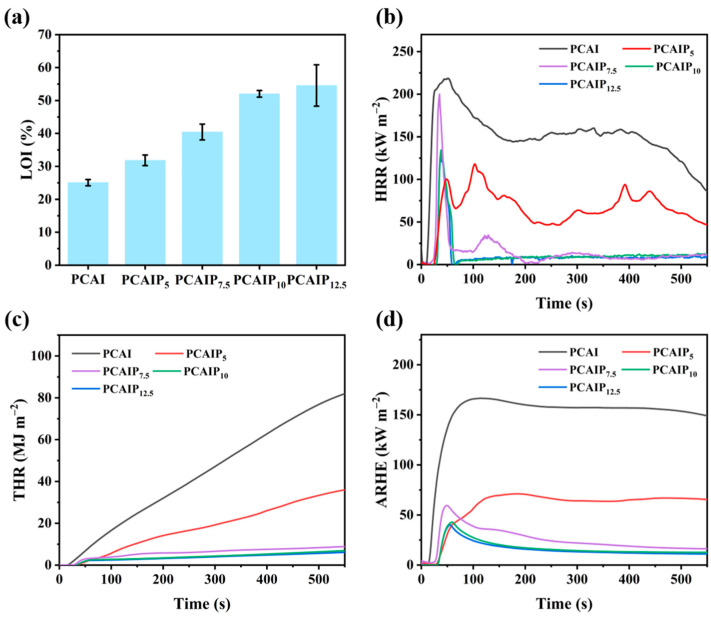
(**a**) LOI values of PCAI and PCAIP_X_. (**b**) HRR, (**c**) THR, and (**d**) ARHE curves of PCAI and PCAIP_X_ from CCT.

The limiting oxygen index (LOI) serves as a pivotal parameter extensively employed in industry to assess the flame resistance of plastics. It is inferred that the LOI values correlate closely with the P content. The PCAI and PCAIP_X_ samples were tested for UL-94 and LOI to evaluate the flame retardancy and the testing results are presented in Figure 3a and Table 1. The LOI value of pure PCAI was only 25.03%, which shows a certain flame retardancy property due to the presence of N-containing ChCl. The values of the LOI increased from 25.03% for PCAI to 31.83%, 40.43%, 52.03%, and 54.57% for PCAIP_5_, PCAIP_7.5_, PCAIP_10_, and PCAIP_12.5_, respectively. Compared with PCAI, the LOI value of PCAIP_12.5_ increased by approximately 118%. The LOI values of PCAIP_X_ obviously increased with the loading of PA, effectively suppressing its combustion.

Cone calorimetry tests (CCT) were conducted to further elucidate the influence of PA on the combustion characteristics of PCAIP_X_. Unlike the LOI determination and UL-94 tests, which provided limited information, CCT offered a comprehensive suite of data regarding combustion behavior, enabling a holistic evaluation of the thermal performance and fire hazard potential of PCAIP_X_. The heat release rate (HRR), total heat release (THR), and average rate of heat emission (ARHE) curves for PCAIP_X_ are presented in Figure 3b–d, with detailed data summarized in Table 2. PCAI exhibited a pHRR of 225.9 kW/m^2^. Upon incorporation of PA, the pHRR decreased to 199.9 kW/m^2^ for PCAIP_5_, indicating that the addition of 5 wt% PA effectively reduced the pHRR. Furthermore, an increase in PA content led to a more pronounced reduction in pHRR, suggesting that a higher PA content enhances the thermal inhibitory capacity of PCAIP_X_. However, the rate of decline in pHRR diminished with further increases in PA content. Concurrently, the THR and pARHE values decreased with the addition of PA (see Figure 3c,d), highlighting the significant role of the P-containing structure of PA in reducing the heat release of PCAI. As shown in Table 2, the TTI of PCAIP_X_ gradually increased with increasing PA content, rising from 19 s for PCAI to 34 s for PCAIP_5_. Overall, the incorporation of PA effectively suppressed the combustion heat release of PCAI, thereby enhancing its flame retardancy. This improvement is attributed to the phosphorus within the PCAIP_X_ structure, which generates phosphoric acid and polyphosphoric acid compounds during combustion. These compounds promote the dehydration and carbonization of PCAIP, leading to the formation of a stable char layer. Additionally, the presence of nitrogen within the char layer enhances its thermal stability and mechanical strength [10,19]. During combustion, nitrogen forms nitrogen–carbon bonds, reinforcing the cross-linking structure of the char layer and reducing its susceptibility to decomposition at elevated temperatures. This char layer acts as a barrier to oxygen and heat, minimizing contact between the material’s interior and the flame, thereby decelerating the combustion rate. Moreover, as the PA content in the PCAIP_X_ structure increases, its flame-inhibiting, charring, and barrier effects become more pronounced. This suggests that higher phosphorus content in the PCAIP_X_ structure is more conducive to enhancing char formation and improving the quality of residual char in PCAI.

**Figure 4:**
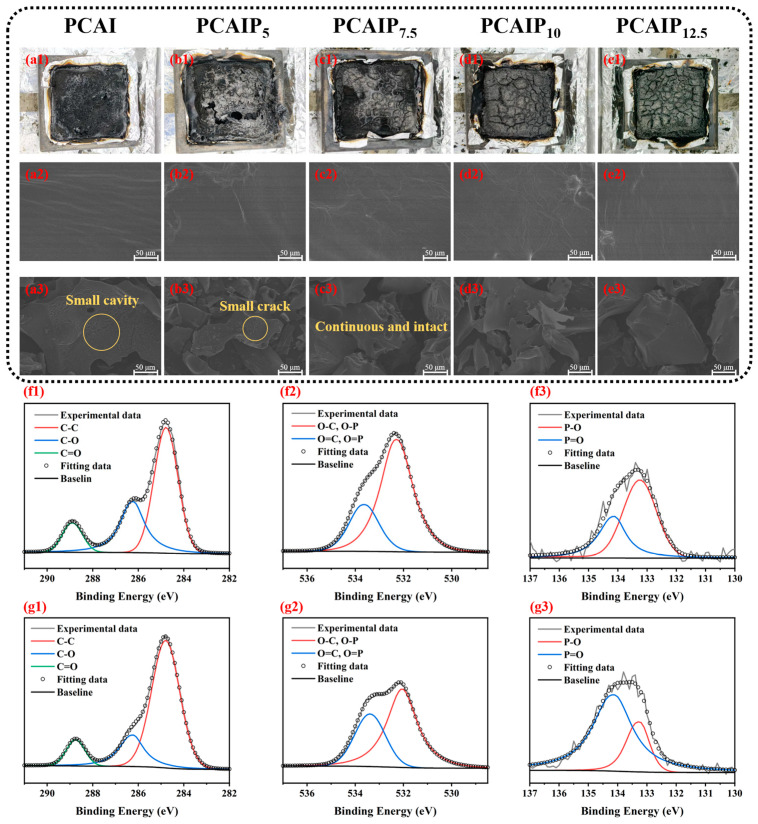
(**a1**–**e1**) Digital images of char residues. SEM images of the pristine samples (**a2**–**e2**) as well as of char residues (**a3**–**e3**) after CCT of PCAI and PCAIPx. XPS of the pristine samples (**f1**–**f3**) and of char residues (**g1**–**g3**) after CCT of PCAIPx.

The flame-retardant mechanism of PA in the condensed phase of PCAIP_X_ was investigated by examining the morphology of the residual char following CCT. Digital photographs of the exterior of the residual char and SEM images of the PCAIP_X_ samples are presented in Figure 4. Examination of the exterior surface of the residual char revealed that the pure PCAI exhibited a fragmented char layer (Figure 4a1), with numerous pores and a thin char height of less than 0.5 cm, such that the underlying tin foil was partially visible. In contrast, as the PA content increased, the char layer became progressively denser and taller (Figure 4b1–e1), with a height approaching 2.4 cm for the PCAIP_X_ sample. This enhancement in char density and height was attributed to the expansion effect and catalytic char-forming properties of PA. SEM analysis further revealed that, while the surfaces of the samples were smooth (Figure 4a2), the char layer of pure PCAI contained a higher density of pores (Figure 4a3). Conversely, the introduction of PA resulted in a denser and more continuous char layer with fewer pores (Figure 4b3–e3). The formation of this robust, dense char layer effectively acted as a barrier, preventing further decomposition of the underlying resin, impeding the transfer of heat and mass, and isolating the oxygen necessary for combustion. The XPS curves of PCAIP_7.5_ before and after combustion are shown in Figure 4f1–g3. The C1s spectra before and after combustion both show three peaks, corresponding to C=O, C-O and C-C, respectively. Their peak area ratio changes from 1:0.52:0.20 before combustion to 1:0.27:0.14 after combustion, indicating that more carbonized layers are formed after combustion, which is beneficial to the flame-retardant effect. Two peaks can be observed in the O1s spectrum; the peaks at 532.2 eV and 533.6 eV belong to C-O-, C-O-C and C=O, P=O, respectively, and the peak area ratio decreases from 1:0.36 before combustion to 1:0.58. In Figure 4f3,g3, two peaks can be observed at 133.25 eV and 134.12 eV, corresponding to P-O-C and P=O, respectively. The peak area ratio of P=O to P-O increases from 0.52:1 before combustion to 1:0.29. This is because the P=O bond has a high bond energy and is relatively stable. Under the high-temperature environment of combustion, the phosphorus-containing PCAIP_7.5_ undergoes thermal decomposition, and chemical bonds such as the P-O-C bond may be broken to form intermediate products such as phosphoric acid, metaphosphoric acid, and polyphosphoric acid. These phosphorus-containing compounds will further dehydrate to form substances such as polyphosphoric acid with a strong dehydrating effect, which promotes the formation of a carbonized layer on the surface of the combustible. In this process, phosphorus atoms combine with oxygen atoms to form P=O bonds, existing in the form of phosphoric anhydride and other forms, thus playing a flame-retardant role. This result is consistent with the increase in the area ratio of C=O and P=O.

Based on the results obtained from CCT, SEM, and XPS analyses, the proposed flame-retardant mechanism of PCAIP_X_ are as follows: PCAIP_X_ exerts its flame-retarding effects in both the gas and condensed phases. During the gas-phase combustion process, certain P-containing compounds decomposed from PA are capable of scavenging H and OH radicals in the combustion region. This radical-trapping action effectively inhibits the chain-reaction of burning, thereby suppressing the combustion process. PCAIP_X_, upon thermal decomposition, generates non-flammable gases such as NH_3_, H_2_O, and NO_2_. These gases act to dilute the concentration of combustible gases in the vicinity of the combustion zone. By reducing the proportion of combustible gases, the likelihood and intensity of combustion are decreased. The polyphosphoric/phosphoric acid, which is derived from the pyrolysis of PA in PCAIP_X_, plays a crucial role in the condensed-phase flame-retardant mechanism. It promotes the carbonization of the PCAIP_X_ elastomer. As a result, the quality and compactness of the char layer formed on the surface of the PCAIP_X_ elastomer are enhanced. This high-quality char residue serves as a physical barrier, impeding the transmission of heat from the flame to the internal elastomer. Additionally, it prevents the release of combustible gases from the substrate, thus avoiding the continued decomposition of the inner material and further suppressing the combustion process [4,9,18,36].

### 2.3. Mechanical Property

**Figure 5:**
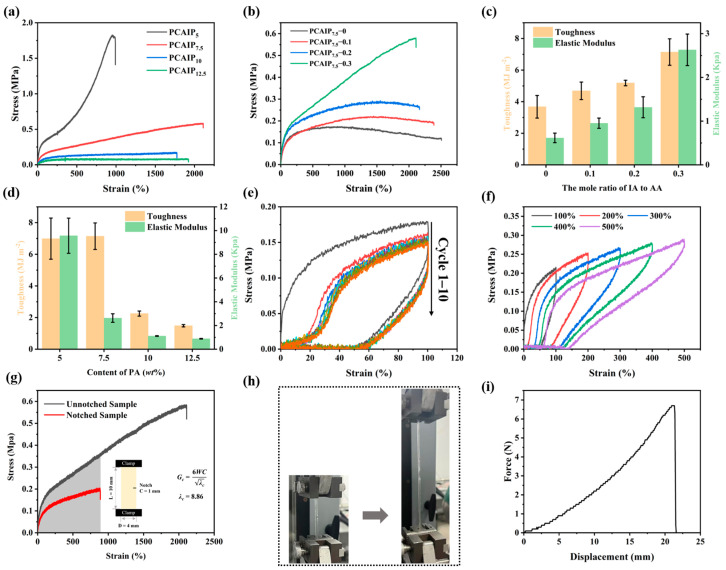
(**a**) Stress–strain curves of PCAIP_x_ elastomers with different PA contents. (**b**) Stress–strain curves of PCAIP_7.5_ elastomers with different IA contents. (**c**) Young’s modulus and toughness of PCAIP_7.5_ elastomers with different IA contents. (**d**) Young’s modulus and toughness of PCAIP_x_ elastomers with different PA contents. (**e**) Stress–strain curves of PCAIP_7.5_-0.3 subjected to cyclic loading–unloading tests. (**f**) Stress–strain curves of PCAIP_7.5_ upon being stretched to 100% strain without intervals. (**g**) Stress–strain curves of unnotched and notched PCAIP_7.5_-0.3. (**h**) Digital image of stretching test. (**i**) Force–displacement curves of PCAIP_7.5_-0.3 in the puncture test.

Figure 5a displays the stress–strain curves of PCAIP_X_ elastomers with different PA contents. As the PA content increases, the tensile strength gradually decreases, while the elongation at break increases. This trend is attributed to the increased water content and weakened intermolecular interactions associated with higher PA levels, which facilitate greater molecular mobility. Specifically, the elastomer with a PA content of 7.5% achieves a tensile strength of 0.6 MPa and an elongation at break of 2109%. Figure 5b presents the stress–strain curves of PCAIP_7.5_ elastomers with varying IA contents. It is evident from the figure that the introduction of a small amount of IA enhances the mechanical property of elastomers. For instance, the tensile strength of PCAIP_7.5_-0.1 reaches 0.2 MPa, with an elongation at break of 2387%. Meanwhile, PCAIP_7.5_-0.3 exhibits a tensile strength of 0.6 MPa and an elongation at break of 2180%, which are substantially higher than those of PCAIP_7.5_-0 (0.1 MPa). As shown in Figure 5c, the toughness of the elastomer increases from 3.7 MJ/m^2^ for PCAIP_7.5_-0 to 7.1 MJ/m^2^ for PCAIP_7.5_-0.3, while the elastic modulus rises from 0.6 kPa to 2.6 kPa with increasing IA content. This enhancement is attributed to the strong hydrogen bonding interactions between the carboxyl groups of IA and the polymer chains. Furthermore, Figure 5d indicates that the toughness of the elastomers decreases with increasing PA content. However, PCAIP_7.5_ demonstrates the best toughness, as PA can also form hydrogen bonds with the polymer backbone, thereby contributing to its excellent mechanical properties. Nevertheless, as the PA content further increases, the water content rises, leading to weakened intermolecular interactions and enhanced molecular mobility, which ultimately results in reduced mechanical properties. Therefore, PCAIP_7.5_-0.3 was selected for subsequent applications and tests in this study.

In the application of ICE material, durability is one of the key parameters in addition to mechanical properties. Therefore, cyclic tensile tests were conducted on PCAIP_X_ elastomers to investigate their fatigue resistance. Figure 5e presents the typical tensile loading–unloading curves of the PCAIP_7.5_-0.3 elastomer measured at different strains (100%, 200%, 300%, 400%, and 500%). The stress–strain curves exhibit pronounced hysteresis loops, indicating the reversible dissociation of dynamic hydrogen bonds within the elastomer, which dissipates a portion of the stretching energy. Additionally, continuous tensile loading–unloading tests were performed on PCAIP_7.5_-0.3 at a fixed strain of 100% to study its viscoelastic behavior under this condition, with results shown in Figure 5f. It can be observed that, although hysteresis loops are still evident, the stress loss does not significantly diminish after five cycles of stretching, suggesting that the elastomer possesses favorable recovery characteristics.

Tear resistance is another critical mechanical property of ICEs. High tear resistance enables elastomers to withstand significant loads without continuous degradation. To characterize its tear resistance, notched tensile tests were conducted to evaluate the ability of PCAIP_X_ to resist tearing in the presence of defects and damage (Figure 5g,h). The tear energy was calculated using the Greensmith method [47,48]. The results indicate that, even with a notch of 1 mm, the PCAIP_7.5_-0.3 elastomer exhibits a tear elongation at break of 886% and a tear strength of 0.2 MPa. Additionally, its tear energy is as high as 4.47 kJ/m^2^. This is attributed to the dynamic dissociation and reformation of hydrogen bonds between the carboxyl groups of IA and PA. The molecular chains of the polymer undergo stick–slip motion under the influence of external forces, effectively transferring the stress at the crack tip to the entire polymer elastomer network and preventing lateral crack propagation. A puncture needle with a diameter of 1 mm was also used to compress the PCAIP_7.5_-0.3 at a rate of 200 mm/min until the sample with a thickness of 0.4 mm was completely penetrated. The PCAIP_7.5_-0.3 exhibits nice puncture resistance. As can be seen from the obtained puncture force–displacement curves in Figure 5i, the puncture force of PCAIP_7.5_-0.3 is determined to be 6.7 N, and the maximum displacement reaches 21 mm.

### 2.4. Adhesion Performance

**Figure 6:**
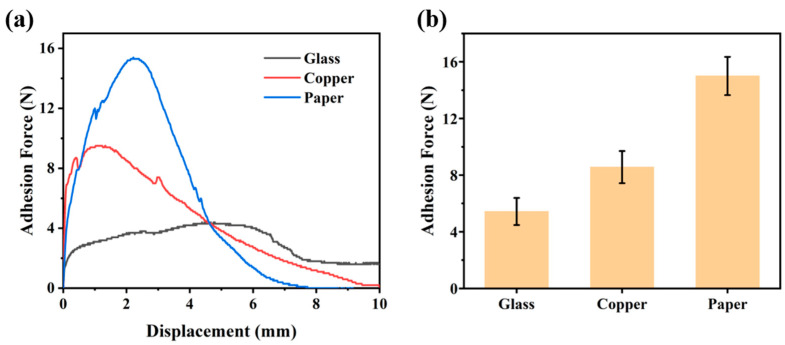
(**a**) Adhesion strength curves and (**b**) adhesion strength values of PCAIP_7.5_-0.3 on different substrates.

The PCAIP_X_ elastomer network is rich in -COOH, -OH, and zwitterionic groups, which endow the material with excellent adhesive properties. We evaluated the adhesion of the PCAIP_7.5_-0.3 elastomer to various substrates, including glass, copper, and paper. To quantify the adhesion strength between the elastomer and these three substrates, the PCAIP_7.5_-0.3 elastomer was assembled between the substrates and subjected to overlap shear tests using a universal tensile machine. The adhesion curves are shown in Figure 6a. The PCAIP_7.5_ elastomer exhibited the highest adhesion force to paper (15 N), followed by copper (8.5 N), and the lowest to glass (5.4 N). The adhesion between the PCAIP_7.5_-0.3 elastomer and the various substrates is attributed to the non-covalent interactions between the functional groups on the substrate surfaces and the abundant −OH, −COOH, and zwitterionic groups within the elastomer network. The ability of the PCAIP_7.5_-0.3 elastomer to readily adhere to a wide range of substrates facilitates its application in the field of sensors.

### 2.5. Application of PCAIP_X_ Elastomers as Strain Sensors

**Figure 7:**
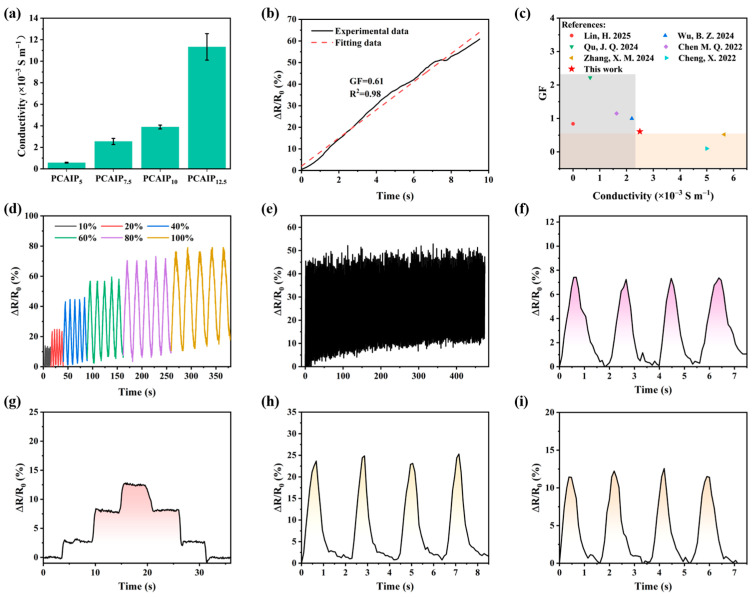
(**a**) Ionic conductivity of PCAIP_x_ elastomers with different PA contents. (**b**) Changes in the relative resistance of the sensor with variations in strains. (**c**) GF and ionic conductivity compared with values from the literature [51,52,53,54,55,56]. (**d**) Real-time monitoring of relative resistance at different strains. (**e**) Cyclic stability test of the sensor under 50% strain for 100 cycles. Real-time monitoring of repeated motions of (**f**) finger, (**g**) wrist, (**h**) elbow, and (**i**) knee by the PCAIP_7.5_-based sensor.

The PCAIP_X_ elastomers exhibit adjustable ionic conductivity and elasticity, endowing them with strain-sensitive electrical response behavior. As shown in Figure 7a, with the increase in the content of PA, its ionic conductivity shows a distinct increasing trend. This is because the increase in the content of PA leads to an increase in the water content, which promotes the ionization of ChCl and further enhances the ionic conductivity. A simple strain sensor was fabricated by connecting the ends of the PCAIP_7.5_ elastomer to conductive copper wires using 3M insulating tape. As depicted in Figure 7b, the relative resistance of the sensor gradually increased with increasing tensile strain. The gauge factor (GF) was calculated to be 0.61 over the strain range of 0–100%, indicating the elastomer’s high sensitivity to strain. The PCAIP_7.5_ exhibited better performance than other ion-conducting elastomers in terms of both GF and ionic conductivity, which is shown in Figure 7c. Additionally, the simple strain sensor constructed from PCAIP_7.5_ was subjected to cyclic tensile sensing tests at various strains (10–100%). As shown in Figure 7d, the relative change in resistance increased with increasing strain. Moreover, stability is one of the critical criteria for evaluating sensor materials. The PCAIP_7.5_ elastomer was tested for 100 cycles at a strain of 50%. As illustrated in Figure 7e, the overall trend of the curve remained unchanged throughout the 100 cycles, demonstrating the elastomer’s favorable cyclic stability. The strain sensor based on this elastomer was employed to monitor human body movements in real time. The detection results shown in Figure 7f–i reveal that the sensor exhibited repeatable and stable resistance change signals in response to repetitive bending motions of the wrist, fingers, elbow, and knee. This indicates that the PCAIP_X_-based sensor has a high recognition capability for movements of different body parts. The above results suggest that the conductive elastomer based on PCAIP_X_ is a promising material for the fabrication of intelligent sensors and holds significant potential for applications in human motion monitoring and related fields.

## 3. Materials and Methods

### 3.1. Materials

Choline chloride (ChCl, 99%, Adamas-Beta, Shanghai, China), acrylic acid (AA, 99% Aladdin, Shanghai, China), itaconic acid (IA, 99%, Aladdin), phytic acid (PA, 50% aqueous solution, Macklin, Shanghai, China), and 2-hydroxy-2-methylpropiophenone (photoinitiator 1173, 97%, Macklin, Shanghai, China) were used as received if not otherwise mentioned.

Software and version used: Origin 2024, Office 365, TH2832, Trios 5.1.1.

### 3.2. Synthesis of Phosphorus–Nitrogen-Containing PCAIP_X_ Elastomers

#### 3.2.1. Preparation of ChCl/AA/IA (CAI) and ChCl/AA/IA/PA (CAIP_X_) PDES

Before use, ChCl was recrystallized from ethanol, AA was dried over a 4 Å molecular sieve, and IA as the hydrogen bond donor was dried under vacuum at 50 °C for 2 h. ChCl, AA, and IA were added to a round-bottom flask at a molar ratio of 1:2:0.3, and then PA with different mass fractions was added. The obtained mixture was heated at 90 °C and stirred for 1 h, and a transparent and colorless PDES could be obtained. The obtained PDES was named CAIP_x_, where x represents the mass fraction of PA. The PDES prepared from ChCl, AA, and IA at a molar ratio of 1:2:0.3 using the same method as above was named CAI.

To verify the effects of different contents of IA in the polymer, while keeping the molar ratio of ChCl to AA at 1:2 and the mass fraction of PA at 7.5 wt%, PDESs with different IA contents were prepared using the same method. The obtained PDESs were named PCAI_7.5_-Y, where Y represents the molar ratio of IA to ChCl.

#### 3.2.2. Preparation of Elastomers via Photopolymerization of CAI and CAIP_X_

The following typical procedure was used for preparing PCAI and PCAIP_X_ elastomers: 0.1 mol% (relative to AA and IA monomers) photoinitiator 1173 was added to the above obtained PDES and stirred for 30 min at room temperature until a clear and transparent colorless solution was formed. The resulting solution was then poured into a polytetrafluoroethylene mold with dimensions of 4 cm × 2 cm × 0.1 cm (length × width × thickness) and irradiated under a UV lamp (B-100AP, UVP Analytik Jena, Jena, Germany) with a wavelength of 365 nm for 10 min. The light intensity was measured to be 10 mW cm^−2^ using a UV radiometer (UV-A, Beijing Shida Photoelectric Technology, Beijing, China). The obtained products were named PCAIP_X_, where x represents the mass fraction of PA. The product prepared by the photopolymerization of CAI without PA was named PCAI. The elastomers prepared by the photopolymerization of CAIP_7.5_-Y were named PCAIP_7.5_-Y, where Y represents the molar ratio of IA to ChCl. The detailed contents used for preparing the ICEs are listed in Table 3.

### 3.3. Characterization

#### 3.3.1. Fourier Transform Infrared Spectroscopy (FT-IR)

The structural characterization of the obtained products was carried out on an FT-IR instrument (Vertex70, Bruker, Billerica, MA, USA). The samples to be tested were directly placed on the diamond reflection crystal, and the test was carried out in the ATR total reflection mode. The scanning range was 600–4000 cm^−1^, the spectral resolution was 4 cm^−1^, and the number of scans was 32.

#### 3.3.2. Thermogravimetry Analysis (TGA)

TGA was performed on an instrument (Discovery, TA, New Castle, DE, USA) with about 5 mg of the sample in the platinum plate. Beginning with equilibrating at 25 °C, the temperature was ramped from 25 to 700 °C at a heating rate of 10 °C/min under a nitrogen atmosphere.

#### 3.3.3. Differential Scanning Calorimeter (DSC)

DSC curves were obtained using a DSC 2010 instrument (TA, New Castle, DE, USA) from −80 to 100 °C at a heating rate of 10 °C/min with a 5 min isothermal hold at the maximum and minimum temperatures. All glass transition temperatures (*T*_g_) of the samples were reported according to the second heating scans.

#### 3.3.4. Mechanical Tests

The mechanical tests were performed at room temperature on a tensile testing machine (Instron 5965, Missouri S&T, Rolla, MO, USA, 5 kN load cell).

The fracture energy test was conducted using the Greensmith method from a single-edge notched sample with a crack length of 1 mm at room temperature on a tensile testing machine (Instron 5965, 5 kN load cell). The tensile speed was set to 50 mm/min, and the size of samples with and without notches was 12 mm × 2.0 mm × 0.5 mm. The testing condition was at room temperature (25 °C). The fracture energy (*G_C_*) was calculated by the following equation:

$$G_{C}=\frac{6WC}{\sqrt{\lambda_{c}}}$$

where *C* represents the length of the notch (1 mm), *λ_C_* represents the fracture elongation of the notched sample, and *W* represents the strain energy calculated by integrating the stress–strain curve of the unnotched sample until *ε_C_* (*ε_C_* = *λ_C_* − 1).

#### 3.3.5. Puncture Resistance Test

The film sample with a thickness of about 0.4 mm was fixed between two rectangular metal sheets. The film deformed freely during the compression process, carried by a metal needle with a diameter of 1 mm. The needle was positioned perpendicularly and moved down to the film in a compression machine equipped with a compression speed of 200 mm/min.

#### 3.3.6. Electrochemical Impedance Spectroscopy (EIS)

EIS was applied using an electrochemical workstation (CS350, Corrtest, Wuhan, China) with a test frequency range of 1 Hz–100 kHz. An elastomer film and two symmetrical stainless-steel electrodes (SS, Φ = 14 mm) were assembled into a blocking cell (SS/elastomer/SS). The ionic conductivity (σ) of the elastomer was calculated according to the following equation:

σ = L/(R × S)

where R is the bulk resistance, L is the thickness of the elastomer (0.1 cm), and S is the contact area of the sample and electrodes.

#### 3.3.7. Scanning Electron Microscope (SEM)

The morphologies of the of the samples and char residues were observed by an SEM instrument (Hitachi SU8010, Hitachi, Tokyo, Japan). Prior to observation, both the sample and char residue were subjected to gold sputtering under conditions of 10 mA current for 60 s. The SEM was operated at an accelerating voltage of 5 kV for imaging.

#### 3.3.8. Flame-Retardant Test

The limiting oxygen index (LOI) values of the material were measured on an instrument (SHI5706A, Guangzhou Xinhe Electronic Equipment, Guangzhou, China) using a 10 cm × 1 cm × 0.4 cm standard sample fixed in the oxygen index tester according to the requirements of GB/T 2406.2 [57]. Cone calorimetric testing (CCT) of the elastomers with a square sheet of 10 cm × 10 cm × 0.3 cm was performed on an instrument (FTT iCons Classic, FTT, Derby, UK), and the combustion test was conducted in the cone calorimeter with a radiant power of 35 kW/m^2^. We conducted three tests and took the average value of the LOI.

#### 3.3.9. Sensing Performance Test

Sensing performances were measured using an LCR meter (TH2830, Changzhou Tonghui Electronics, Changzhou, China) at a frequency of 1.0 kHz and a constant voltage of 1.0 V. Two copper wires were connected to the test apparatus, and the other ends of the two copper wires were connected to each end of the elastomer (4 cm × 2 cm × 0.1 cm). Cyclic tensile sensing tests were performed using a tensile machine (Instron 5965, 5 kN load cell) to record the change in relative resistance signal with strain in real time. The testing condition was at room temperature (25 °C).

#### 3.3.10. X-Ray Photoelectron Spectroscopy (XPS) Test

XPS was performed on an EXCALAB 250 XI (Thermo Scientifc, Waltham, MA, USA). The energy resolution error was less than 0.5 eV, and the sensitivity was better than 400 KCPS.

## 4. Conclusions

This work designed and synthesized a series of flame-retardant ICEs (PCAIP_x_) with multiple hydrogen bonds with adjustable chemical structures and P content. The incorporation of PA effectively rendered PCAIP_x_ with good fire safety attributes, leading to the attainment of a UL-94 V-0 rating of all PCAIP elastomers. PCAI exhibited a pHRR of 225.9 kW/m^2^, and, when PA was subjected to the PCAI, the value of pHRR was 199.9 kW/m^2^ for the PCAIP_5_. The incorporation of 5% PA proved effective in diminishing the pHRR of PCAI. SEM images of the char layer of the pure PCAI contained more holes, while, with the introduction of PA, the char layer became denser with fewer holes. PCAIP_7.5_-0.3 exhibited a tensile strength of 0.6 MPa and an elongation at break of 2180%, which were higher than those of PCAIP_7.5_-0 (0.1 MPa). The stress–strain curves exhibit pronounced hysteresis loops, indicating the reversible dissociation of dynamic hydrogen bonds within the elastomer, which dissipates a portion of the stretching energy. The PCAIP_7.5_ elastomer exhibited a tear elongation at break of 886% and a tear strength of 0.2 MPa and exhibited the highest adhesion force to paper (15.0 N), followed by copper (8.6 N), and the lowest to glass (5.4 kPa). The PCAIP_7.5_ elastomer exhibited favorable ionic conductivity and elasticity, which endow it with strain-sensitive electrical response behavior. The detection results revealed that the sensor exhibited repeatable and stable resistance change signals in response to repetitive bending motions of the wrist, fingers, elbow, and knee. In conclusion, these flame-retardant ICEs hold potential for applications in electronics, sports equipment, and aviation.

## Figures and Tables

**Table 1:** Flame retardancy of PCAI and PCAIP_X_.

Samples	PA (wt%)	UL-94 Test		LOI (%)
		Dripping/Ignition of Cotton	Rating	
PCAI	0	Yes/Yes	V-2	25.0 ± 1.0
PCAIP_5_	5	No/No	V-0	31.8 ± 1.6
PCAIP_7.5_	7.5	No/No	V-0	40.4 ± 2.4
PCAIP_10_	10	No/No	V-0	52.0 ± 1.0
PCAIP_12.5_	12.5	No/No	V-0	54.6 ± 6.3

**Table 2:** Detailed results of PCAI and PCAIP_X_ from CCT.

Samples	TTI ^a^ (s)	pHRR ^b^ (kw/m^2^)	THR ^c^ (MJ/m^2^)	pARHE ^d^ (kw/m^2^)	TML ^e^ (g/m^2^)
PCAI	19	225.9	93.1	166.5	5170
PCAIP_5_	34	199.9	44.2	71.1	3314
PCAIP_7.5_	35	134.4	25.5	59.4	3263
PCAIP_10_	36	130.4	15.1	42.8	3190
PCAIP_12.5_	37	120.5	15.3	40.0	3187

^a^ Time to ignition; ^b^ the peak of heat release rate; ^c^ total heat release; ^d^ the peak of the average rate of heat emission; ^e^ total mass loss.

**Table 3:** Detailed contents used for preparing ICEs.

Samples	m (ChCl, g)	m (AA, g)	m (IA, g)	m (PA, g)
PCAI	4.84	5	1.35	0
PCAIP_5_				1.12
PCAIP_7.5_				1.68
PCAIP_10_				2.24
PCAIP_12.5_				2.80
PCAIP_7.5_-0			0	1.48
PCAIP_7.5_-0.1			0.45	1.54
PCAIP_7.5_-0.2			0.90	1.61
PCAIP_7.5_-0.3			1.35	1.68

## Data Availability

The original contributions presented in this study are included in the article. Further inquiries can be directed to the corresponding authors.
